# [*N*,*N*′-Bis(3-meth­oxy-2-oxidobenzyl­idene)ethyl­enediammonium-κ^4^ *O*,*O*′,*O*′′,*O*′′′]tris­(nitrato-κ^2^ *O*,*O*′)dysprosium(III)

**DOI:** 10.1107/S1600536809047436

**Published:** 2009-11-14

**Authors:** Ting Gao, Guang-Ming Li, Po Gao, Peng-Fei Yan, Guang-Feng Hou

**Affiliations:** a School of Chemistry and Materials Science, Heilongjiang University, Harbin 150080, People’s Republic of China

## Abstract

In the title mononuclear Schiff base complex, [Dy(NO_3_)_3_(C_18_H_20_N_2_O_4_)], the Dy^III^ ion is ten-coordinated in a distorted hexa­deca­hedral geometry by six O atoms of three nitrate anions and four O atoms of the Schiff base ligand. An intra­molecular N—H⋯O hydrogen bond occurs. The crystal structure is stabilized by inter­molecular C—H⋯O hydrogen bonds.

## Related literature

For the synthesis and crystal structure of the isostructural Nd, Eu and Tb complexes, see: Gao *et al.* (2008 ▶).
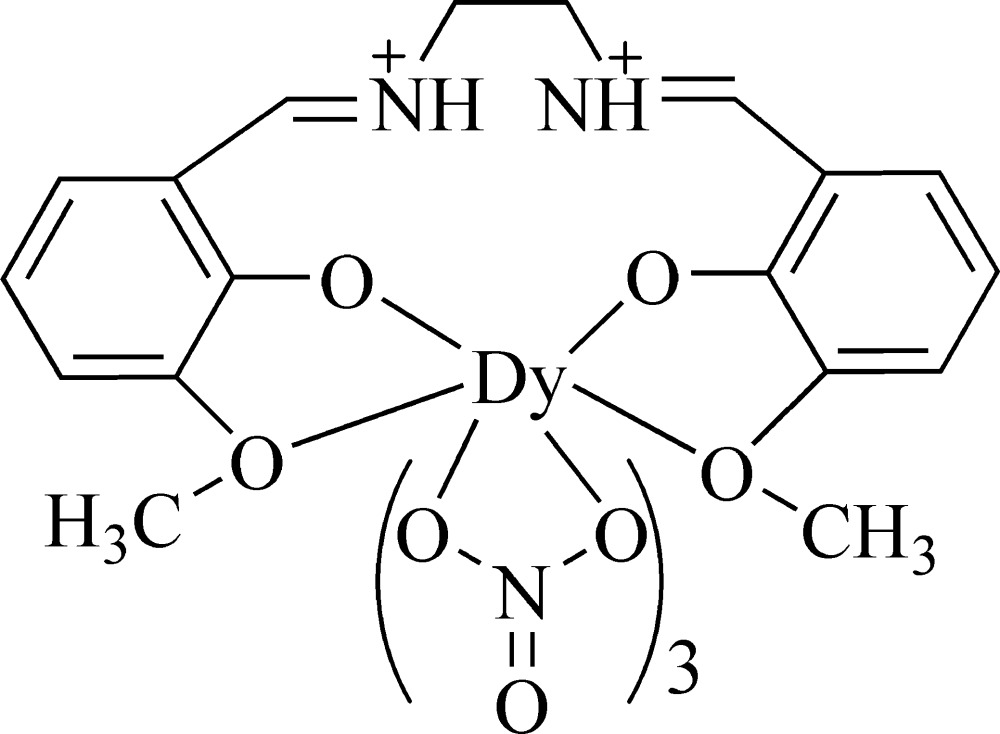


## Experimental

### 

#### Crystal data

[Dy(NO_3_)_3_(C_18_H_20_N_2_O_4_)]
*M* *_r_* = 676.89
Monoclinic, 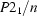

*a* = 14.126 (5) Å
*b* = 11.860 (4) Å
*c* = 14.628 (4) Å
β = 104.302 (12)°
*V* = 2374.7 (13) Å^3^
*Z* = 4
Mo *K*α radiation
μ = 3.22 mm^−1^
*T* = 291 K
0.29 × 0.28 × 0.24 mm

#### Data collection

Rigaku R-AXIS RAPID diffractometer
Absorption correction: multi-scan (*ABSCOR*; Higashi, 1995 ▶) *T*_min_ = 0.454, *T*_max_ = 0.513
22587 measured reflections
5430 independent reflections
4850 reflections with *I* > 2σ(*I*)
*R*_int_ = 0.021

#### Refinement

*R*[*F* ^2^ > 2σ(*F* ^2^)] = 0.020
*wR*(*F* ^2^) = 0.045
*S* = 1.10
5430 reflections
344 parameters
14 restraints
H atoms treated by a mixture of independent and constrained refinement
Δρ_max_ = 0.41 e Å^−3^
Δρ_min_ = −0.54 e Å^−3^

### 

Data collection: *RAPID-AUTO* (Rigaku, 1998 ▶); cell refinement: *RAPID-AUTO*; data reduction: *CrystalStructure* (Rigaku/MSC, 2002 ▶); program(s) used to solve structure: *SHELXS97* (Sheldrick, 2008 ▶); program(s) used to refine structure: *SHELXL97* (Sheldrick, 2008 ▶); molecular graphics: *SHELXTL* (Sheldrick, 2008 ▶); software used to prepare material for publication: *SHELXL97*.

## Supplementary Material

Crystal structure: contains datablocks global, I. DOI: 10.1107/S1600536809047436/rz2383sup1.cif

Structure factors: contains datablocks I. DOI: 10.1107/S1600536809047436/rz2383Isup2.hkl

Additional supplementary materials:  crystallographic information; 3D view; checkCIF report

## Figures and Tables

**Table 1:** Selected bond lengths (Å)

Dy1—O1	2.2718 (18)
Dy1—O3	2.2847 (18)
Dy1—O10	2.472 (2)
Dy1—O8	2.477 (2)
Dy1—O5	2.480 (2)
Dy1—O13	2.490 (2)
Dy1—O11	2.492 (2)
Dy1—O7	2.510 (2)
Dy1—O4	2.5740 (19)
Dy1—O2	2.6794 (19)

**Table 2:** Hydrogen-bond geometry (Å, °)

*D*—H⋯*A*	*D*—H	H⋯*A*	*D*⋯*A*	*D*—H⋯*A*
N2—H2⋯O3	0.850 (15)	1.87 (3)	2.570 (3)	139.3 (14)
C8—H8*B*⋯O12^i^	0.97	2.51	3.245 (3)	133
C10—H10⋯O5^ii^	0.93	2.32	3.076 (3)	138
C3—H3⋯O12^iii^	0.93	2.51	3.351 (3)	151
C7—H7⋯O9^iv^	0.93	2.56	3.376 (3)	147

Symmetry codes: (i) 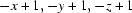; (ii) 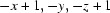; (iii) 
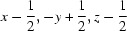
; (iv) 
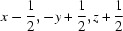
.

**Fig. 1.**
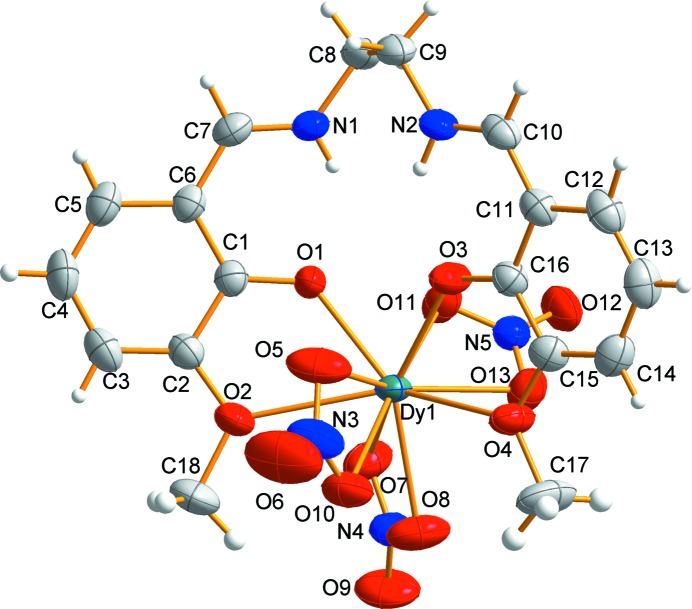
The molecular structure of the title compound, showing 30% probability displacement ellipsoids.

## supplementary crystallographic information

### Comment

Schiff base lanthanide complexes are currently of great interest because of their unique physicochemical properties and various applications as new materials. Moreover, the luminescence and magnetic properties of lanthanide complexes have recently aroused much attention.

As shown in Fig. 1, the ten-coordinate dysprosium(III) ion adopts a hexadecahedron geometry provided by the O atoms of three bidentate nitrate anions and by one ligand that utilizes two hydroxyl and two methoxy oxygen atoms, while the protonated nitrogen atoms remain uncoordinated. The title compound is isostructural with the corresponding Nd, Eu and Tb complexes (Gao *et al.*, 2008). The Dy—O bond distances (Table 1) range from 2.2718 (18) to 2.6794 (19) Å, the shorter bonds involving the O1 and O3 deprotonated phenol oxygen atoms. The crystal structure is stabilized by intra- and intermolecular N—H···O and C—H···O hydrogen bonds (Table 2).

### Experimental

The title complex was obtained by the treatment of dysprosium (III) nitrate hexahydrate (0.114 g, 0.25 mmol) with *N*,*N*'-ethylene-bis(3-methoxysalicylideneimine) (0.083 g, 0.25 mmol) in acetonitrile/methanol (10 ml/10 ml). The mixture was stirred for 3 h. The reaction mixture was then filtered and diethyl ether was allowed to diffuse slowly into the solution of the filtrate. Single crystals were obtained after several days. Analysis calculated for for C_18_H_20_Dy_1_N_5_O_13_: C 31.94, H 2.96, N 10.37%; found: C 32.08, H 3.00, N 10.48%.

### Refinement

H atoms bound to C atoms were placed in calculated positions and treated as riding on their parent atoms, with C—H = 0.93 Å (aromatic), C—H = 0.97Å (methylene), and with *U*_iso_(H) = 1.2*U*_eq_(C) or C—H = 0.96 Å (methly) and with *U*_iso_(H) = 1.5*U*_iso_(C). The N-bound H atoms were initially located in a difference Fourier map and refined with N—H = 0.85 Å. The N3, N4, O5, O7, O8 and O10 atoms were restrained to be nearly isotropic by the ISOR (tolerance 0.01) instruction of SHELXL-97 (Sheldrick, 2008)

### Crystal data

**Table:**

[Dy(NO_3_)_3_(C_18_H_20_N_2_O_4_)]	*F*(000) = 1332
*M**_r_* = 676.89	*D*_x_ = 1.893 Mg m^−^^3^
Monoclinic, *P*2_1_/*n*	Mo *K*α radiation, λ = 0.71073 Å
Hall symbol: -P 2yn	Cell parameters from 20034 reflections
*a* = 14.126 (5) Å	θ = 6.7–55.0°
*b* = 11.860 (4) Å	µ = 3.22 mm^−^^1^
*c* = 14.628 (4) Å	*T* = 291 K
β = 104.302 (12)°	Block, brown
*V* = 2374.7 (13) Å^3^	0.29 × 0.28 × 0.24 mm
*Z* = 4	

### Data collection

**Table:**

Rigaku R-AXIS RAPID diffractometer	5430 independent reflections
Radiation source: fine-focus sealed tube	4850 reflections with *I* > 2σ(*I*)
graphite	*R*_int_ = 0.021
ω scans	θ_max_ = 27.5°, θ_min_ = 3.4°
Absorption correction: multi-scan (*ABSCOR*; Higashi, 1995)	*h* = −18→18
*T*_min_ = 0.454, *T*_max_ = 0.513	*k* = −15→15
22587 measured reflections	*l* = −18→18

### Refinement

**Table:**

Refinement on *F*^2^	Primary atom site location: structure-invariant direct methods
Least-squares matrix: full	Secondary atom site location: difference Fourier map
*R*[*F*^2^ > 2σ(*F*^2^)] = 0.020	Hydrogen site location: inferred from neighbouring sites
*wR*(*F*^2^) = 0.045	H atoms treated by a mixture of independent and constrained refinement
*S* = 1.10	*w* = 1/[σ^2^(*F*_o_^2^) + (0.0164*P*)^2^ + 1.5062*P*] where *P* = (*F*_o_^2^ + 2*F*_c_^2^)/3
5430 reflections	(Δ/σ)_max_ = 0.002
344 parameters	Δρ_max_ = 0.41 e Å^−^^3^
14 restraints	Δρ_min_ = −0.54 e Å^−^^3^

### Special details

**Table:**

Geometry. All e.s.d.'s (except the e.s.d. in the dihedral angle between two l.s. planes) are estimated using the full covariance matrix. The cell e.s.d.'s are taken into account individually in the estimation of e.s.d.'s in distances, angles and torsion angles; correlations between e.s.d.'s in cell parameters are only used when they are defined by crystal symmetry. An approximate (isotropic) treatment of cell e.s.d.'s is used for estimating e.s.d.'s involving l.s. planes.
Refinement. Refinement of *F*^2^ against ALL reflections. The weighted *R*-factor *wR* and goodness of fit *S* are based on *F*^2^, conventional *R*-factors *R* are based on *F*, with *F* set to zero for negative *F*^2^. The threshold expression of *F*^2^ > σ(*F*^2^) is used only for calculating *R*-factors(gt) *etc*. and is not relevant to the choice of reflections for refinement. *R*-factors based on *F*^2^ are statistically about twice as large as those based on *F*, and *R*- factors based on ALL data will be even larger.

### Fractional atomic coordinates and isotropic or equivalent isotropic displacement parameters (Å^2^)

**Table:**

	*x*	*y*	*z*	*U*_iso_*/*U*_eq_	
C1	0.29349 (17)	0.18407 (19)	0.20773 (16)	0.0338 (5)	
C2	0.28896 (19)	0.1407 (2)	0.11666 (17)	0.0384 (5)	
C3	0.2045 (2)	0.0947 (2)	0.06330 (19)	0.0488 (7)	
H3	0.2018	0.0683	0.0028	0.059*	
C4	0.1218 (2)	0.0874 (3)	0.1004 (2)	0.0542 (7)	
H4	0.0646	0.0553	0.0643	0.065*	
C5	0.12392 (19)	0.1264 (2)	0.1879 (2)	0.0469 (6)	
H5	0.0687	0.1201	0.2114	0.056*	
C6	0.20936 (17)	0.1765 (2)	0.24346 (17)	0.0362 (5)	
C7	0.21298 (18)	0.2144 (2)	0.33610 (18)	0.0385 (5)	
H7	0.1585	0.2031	0.3600	0.046*	
C8	0.3034 (2)	0.2907 (2)	0.48885 (18)	0.0452 (6)	
H8A	0.2405	0.3002	0.5034	0.054*	
H8B	0.3391	0.3610	0.5028	0.054*	
C9	0.3598 (2)	0.1974 (2)	0.54950 (18)	0.0463 (6)	
H9A	0.3735	0.2192	0.6154	0.056*	
H9B	0.3205	0.1294	0.5415	0.056*	
C10	0.5321 (2)	0.1420 (2)	0.57900 (17)	0.0407 (6)	
H10	0.5346	0.1328	0.6427	0.049*	
C11	0.61751 (19)	0.1191 (2)	0.54777 (16)	0.0377 (5)	
C12	0.7035 (2)	0.0798 (2)	0.61169 (19)	0.0469 (6)	
H12	0.7033	0.0664	0.6743	0.056*	
C13	0.7860 (2)	0.0617 (2)	0.5824 (2)	0.0495 (7)	
H13	0.8424	0.0375	0.6253	0.059*	
C14	0.78698 (19)	0.0792 (2)	0.4876 (2)	0.0452 (6)	
H14	0.8440	0.0666	0.4682	0.054*	
C15	0.70435 (19)	0.1145 (2)	0.42392 (17)	0.0393 (5)	
C16	0.61780 (18)	0.1388 (2)	0.45268 (16)	0.0364 (5)	
C17	0.7761 (3)	0.1084 (4)	0.2912 (3)	0.0785 (11)	
H17A	0.8281	0.1592	0.3194	0.118*	
H17B	0.7584	0.1192	0.2241	0.118*	
H17C	0.7975	0.0321	0.3052	0.118*	
C18	0.3786 (3)	0.1133 (3)	−0.00104 (19)	0.0633 (9)	
H18A	0.3646	0.0340	−0.0063	0.095*	
H18B	0.4423	0.1268	−0.0109	0.095*	
H18C	0.3307	0.1534	−0.0477	0.095*	
Dy1	0.527482 (8)	0.207652 (10)	0.234090 (7)	0.03310 (4)	
H1	0.3368 (15)	0.274 (2)	0.3653 (19)	0.049 (8)*	
H2	0.452 (2)	0.180 (2)	0.4658 (9)	0.050 (8)*	
N1	0.28886 (15)	0.26398 (19)	0.38914 (15)	0.0389 (5)	
N2	0.45122 (17)	0.1750 (2)	0.52358 (14)	0.0418 (5)	
N3	0.5162 (2)	−0.0351 (2)	0.19797 (19)	0.0643 (7)	
N4	0.57521 (19)	0.3122 (2)	0.07175 (16)	0.0477 (6)	
N5	0.58626 (16)	0.42878 (19)	0.31539 (14)	0.0412 (5)	
O1	0.37477 (11)	0.22948 (15)	0.25585 (11)	0.0371 (4)	
O2	0.37584 (14)	0.15168 (17)	0.09135 (12)	0.0464 (4)	
O3	0.54183 (13)	0.17845 (18)	0.39131 (12)	0.0480 (5)	
O4	0.69309 (13)	0.13026 (18)	0.32846 (12)	0.0483 (5)	
O5	0.47264 (19)	0.01237 (19)	0.25317 (15)	0.0697 (7)	
O6	0.5109 (3)	−0.1369 (2)	0.1860 (2)	0.1026 (10)	
O7	0.56475 (17)	0.02863 (18)	0.15829 (15)	0.0586 (5)	
O8	0.62384 (19)	0.2322 (2)	0.11513 (17)	0.0684 (7)	
O9	0.5925 (2)	0.3528 (2)	0.00129 (17)	0.0767 (7)	
O10	0.50641 (15)	0.3465 (2)	0.10457 (15)	0.0591 (5)	
O11	0.49884 (14)	0.39756 (17)	0.29500 (16)	0.0531 (5)	
O12	0.61122 (16)	0.52094 (17)	0.35117 (14)	0.0565 (5)	
O13	0.64723 (13)	0.35980 (17)	0.29731 (15)	0.0532 (5)	

### Atomic displacement parameters (Å^2^)

**Table:**

	*U*^11^	*U*^22^	*U*^33^	*U*^12^	*U*^13^	*U*^23^
C1	0.0348 (11)	0.0314 (11)	0.0346 (11)	−0.0017 (9)	0.0074 (9)	0.0008 (9)
C2	0.0440 (13)	0.0367 (13)	0.0341 (11)	−0.0039 (11)	0.0091 (10)	−0.0033 (10)
C3	0.0588 (17)	0.0428 (14)	0.0395 (13)	−0.0086 (13)	0.0020 (12)	−0.0061 (11)
C4	0.0464 (15)	0.0521 (16)	0.0559 (16)	−0.0168 (13)	−0.0027 (13)	−0.0037 (14)
C5	0.0362 (13)	0.0471 (15)	0.0557 (16)	−0.0084 (12)	0.0084 (12)	0.0055 (13)
C6	0.0327 (11)	0.0353 (12)	0.0406 (12)	−0.0043 (9)	0.0091 (10)	0.0028 (10)
C7	0.0349 (12)	0.0386 (12)	0.0460 (13)	−0.0003 (10)	0.0176 (10)	0.0031 (11)
C8	0.0478 (14)	0.0504 (15)	0.0433 (13)	−0.0048 (12)	0.0225 (11)	−0.0145 (12)
C9	0.0492 (14)	0.0586 (17)	0.0376 (12)	−0.0106 (13)	0.0231 (11)	−0.0047 (12)
C10	0.0553 (15)	0.0370 (13)	0.0310 (11)	−0.0091 (11)	0.0128 (11)	0.0011 (10)
C11	0.0473 (13)	0.0319 (12)	0.0323 (11)	−0.0022 (10)	0.0069 (10)	0.0017 (10)
C12	0.0585 (17)	0.0394 (14)	0.0372 (13)	−0.0020 (12)	0.0014 (12)	0.0035 (11)
C13	0.0491 (15)	0.0404 (14)	0.0510 (15)	0.0051 (12)	−0.0028 (12)	0.0060 (12)
C14	0.0400 (13)	0.0362 (13)	0.0584 (16)	0.0049 (11)	0.0103 (12)	0.0013 (12)
C15	0.0442 (13)	0.0342 (12)	0.0397 (12)	0.0047 (11)	0.0109 (11)	0.0004 (10)
C16	0.0394 (12)	0.0353 (12)	0.0339 (11)	0.0025 (10)	0.0077 (10)	0.0001 (10)
C17	0.060 (2)	0.118 (3)	0.069 (2)	0.037 (2)	0.0370 (17)	0.013 (2)
C18	0.077 (2)	0.082 (2)	0.0358 (14)	0.0003 (18)	0.0229 (14)	−0.0122 (15)
Dy1	0.03516 (6)	0.03925 (7)	0.02910 (6)	0.00211 (5)	0.01592 (4)	−0.00037 (5)
N1	0.0366 (11)	0.0468 (12)	0.0382 (11)	−0.0022 (9)	0.0185 (9)	−0.0047 (9)
N2	0.0471 (12)	0.0519 (13)	0.0294 (10)	−0.0044 (10)	0.0153 (9)	−0.0019 (9)
N3	0.102 (2)	0.0461 (14)	0.0522 (14)	0.0068 (15)	0.0325 (15)	−0.0015 (12)
N4	0.0601 (14)	0.0470 (13)	0.0439 (12)	−0.0118 (11)	0.0278 (11)	−0.0022 (10)
N5	0.0432 (12)	0.0462 (12)	0.0361 (10)	−0.0041 (10)	0.0136 (9)	−0.0033 (9)
O1	0.0298 (8)	0.0492 (10)	0.0333 (8)	−0.0072 (7)	0.0097 (7)	−0.0093 (7)
O2	0.0515 (11)	0.0581 (12)	0.0320 (8)	−0.0042 (9)	0.0151 (8)	−0.0092 (8)
O3	0.0409 (9)	0.0740 (13)	0.0309 (8)	0.0142 (9)	0.0126 (7)	0.0078 (9)
O4	0.0438 (10)	0.0643 (12)	0.0418 (9)	0.0167 (9)	0.0199 (8)	0.0047 (9)
O5	0.117 (2)	0.0492 (12)	0.0607 (13)	0.0073 (12)	0.0550 (14)	0.0087 (10)
O6	0.162 (3)	0.0501 (14)	0.113 (2)	−0.0049 (16)	0.066 (2)	−0.0142 (15)
O7	0.0759 (14)	0.0541 (12)	0.0556 (12)	0.0009 (11)	0.0345 (11)	−0.0100 (10)
O8	0.0819 (16)	0.0684 (14)	0.0724 (15)	0.0239 (13)	0.0523 (13)	0.0187 (12)
O9	0.1095 (19)	0.0714 (15)	0.0662 (14)	−0.0062 (14)	0.0538 (14)	0.0168 (12)
O10	0.0497 (11)	0.0750 (14)	0.0583 (12)	0.0095 (11)	0.0243 (10)	0.0189 (11)
O11	0.0401 (10)	0.0494 (11)	0.0741 (13)	−0.0049 (9)	0.0221 (9)	−0.0172 (10)
O12	0.0668 (13)	0.0481 (11)	0.0540 (11)	−0.0170 (10)	0.0137 (10)	−0.0135 (9)
O13	0.0377 (9)	0.0549 (12)	0.0677 (13)	0.0011 (9)	0.0142 (9)	−0.0042 (10)

### Geometric parameters (Å, °)

**Table:**

C1—O1	1.306 (3)	C15—O4	1.379 (3)
C1—C6	1.414 (3)	C15—C16	1.417 (3)
C1—C2	1.415 (3)	C16—O3	1.305 (3)
C2—C3	1.367 (4)	C17—O4	1.434 (3)
C2—O2	1.373 (3)	C17—H17A	0.9600
C3—C4	1.409 (4)	C17—H17B	0.9600
C3—H3	0.9300	C17—H17C	0.9600
C4—C5	1.353 (4)	C18—O2	1.436 (3)
C4—H4	0.9300	C18—H18A	0.9600
C5—C6	1.410 (3)	C18—H18B	0.9600
C5—H5	0.9300	C18—H18C	0.9600
C6—C7	1.416 (4)	Dy1—O1	2.2718 (18)
C7—N1	1.299 (3)	Dy1—O3	2.2847 (18)
C7—H7	0.9300	Dy1—O10	2.472 (2)
C8—N1	1.457 (3)	Dy1—O8	2.477 (2)
C8—C9	1.515 (4)	Dy1—O5	2.480 (2)
C8—H8A	0.9700	Dy1—O13	2.490 (2)
C8—H8B	0.9700	Dy1—O11	2.492 (2)
C9—N2	1.458 (3)	Dy1—O7	2.510 (2)
C9—H9A	0.9700	Dy1—O4	2.5740 (19)
C9—H9B	0.9700	Dy1—O2	2.6794 (19)
C10—N2	1.288 (3)	N1—H1	0.843 (10)
C10—C11	1.418 (4)	N2—H2	0.848 (10)
C10—H10	0.9300	N3—O6	1.219 (4)
C11—C16	1.411 (3)	N3—O7	1.255 (3)
C11—C12	1.416 (4)	N3—O5	1.263 (3)
C12—C13	1.355 (4)	N4—O9	1.216 (3)
C12—H12	0.9300	N4—O8	1.248 (3)
C13—C14	1.404 (4)	N4—O10	1.254 (3)
C13—H13	0.9300	N5—O12	1.225 (3)
C14—C15	1.367 (4)	N5—O11	1.253 (3)
C14—H14	0.9300	N5—O13	1.262 (3)
			
O1—C1—C6	122.3 (2)	O3—Dy1—O8	142.89 (8)
O1—C1—C2	119.1 (2)	O10—Dy1—O8	50.68 (7)
C6—C1—C2	118.6 (2)	O1—Dy1—O5	75.92 (7)
C3—C2—O2	126.5 (2)	O3—Dy1—O5	72.40 (7)
C3—C2—C1	120.9 (2)	O10—Dy1—O5	136.61 (8)
O2—C2—C1	112.6 (2)	O8—Dy1—O5	115.47 (7)
C2—C3—C4	119.6 (3)	O1—Dy1—O13	116.54 (6)
C2—C3—H3	120.2	O3—Dy1—O13	81.53 (7)
C4—C3—H3	120.2	O10—Dy1—O13	75.16 (8)
C5—C4—C3	121.1 (2)	O8—Dy1—O13	74.63 (8)
C5—C4—H4	119.4	O5—Dy1—O13	146.88 (8)
C3—C4—H4	119.4	O1—Dy1—O11	66.62 (6)
C4—C5—C6	120.4 (3)	O3—Dy1—O11	76.05 (7)
C4—C5—H5	119.8	O10—Dy1—O11	70.88 (8)
C6—C5—H5	119.8	O8—Dy1—O11	108.11 (8)
C5—C6—C1	119.4 (2)	O5—Dy1—O11	136.15 (7)
C5—C6—C7	120.6 (2)	O13—Dy1—O11	50.77 (6)
C1—C6—C7	119.9 (2)	O1—Dy1—O7	117.92 (7)
N1—C7—C6	123.0 (2)	O3—Dy1—O7	109.89 (7)
N1—C7—H7	118.5	O10—Dy1—O7	102.92 (8)
C6—C7—H7	118.5	O8—Dy1—O7	65.02 (8)
N1—C8—C9	110.6 (2)	O5—Dy1—O7	50.60 (7)
N1—C8—H8A	109.5	O13—Dy1—O7	125.42 (7)
C9—C8—H8A	109.5	O11—Dy1—O7	173.06 (7)
N1—C8—H8B	109.5	O1—Dy1—O4	137.24 (6)
C9—C8—H8B	109.5	O3—Dy1—O4	64.10 (6)
H8A—C8—H8B	108.1	O10—Dy1—O4	124.75 (6)
N2—C9—C8	110.6 (2)	O8—Dy1—O4	80.47 (7)
N2—C9—H9A	109.5	O5—Dy1—O4	82.78 (8)
C8—C9—H9A	109.5	O13—Dy1—O4	67.37 (7)
N2—C9—H9B	109.5	O11—Dy1—O4	109.69 (7)
C8—C9—H9B	109.5	O7—Dy1—O4	70.93 (7)
H9A—C9—H9B	108.1	O1—Dy1—O2	62.19 (6)
N2—C10—C11	123.3 (2)	O3—Dy1—O2	127.01 (6)
N2—C10—H10	118.4	O10—Dy1—O2	69.28 (7)
C11—C10—H10	118.4	O8—Dy1—O2	87.36 (8)
C16—C11—C12	119.6 (2)	O5—Dy1—O2	69.17 (7)
C16—C11—C10	119.7 (2)	O13—Dy1—O2	143.90 (7)
C12—C11—C10	120.6 (2)	O11—Dy1—O2	109.48 (6)
C13—C12—C11	120.5 (3)	O7—Dy1—O2	70.17 (7)
C13—C12—H12	119.7	O4—Dy1—O2	140.83 (6)
C11—C12—H12	119.7	C7—N1—C8	126.0 (2)
C12—C13—C14	120.5 (2)	C7—N1—H1	117 (2)
C12—C13—H13	119.7	C8—N1—H1	117 (2)
C14—C13—H13	119.7	C10—N2—C9	126.7 (2)
C15—C14—C13	120.2 (3)	C10—N2—H2	115 (2)
C15—C14—H14	119.9	C9—N2—H2	118 (2)
C13—C14—H14	119.9	O6—N3—O7	123.5 (3)
C14—C15—O4	126.7 (2)	O6—N3—O5	120.7 (3)
C14—C15—C16	120.9 (2)	O7—N3—O5	115.8 (2)
O4—C15—C16	112.4 (2)	O9—N4—O8	122.1 (3)
O3—C16—C11	122.4 (2)	O9—N4—O10	122.2 (3)
O3—C16—C15	119.5 (2)	O8—N4—O10	115.7 (2)
C11—C16—C15	118.1 (2)	O12—N5—O11	121.9 (2)
O4—C17—H17A	109.5	O12—N5—O13	121.9 (2)
O4—C17—H17B	109.5	O11—N5—O13	116.2 (2)
H17A—C17—H17B	109.5	C1—O1—Dy1	128.06 (15)
O4—C17—H17C	109.5	C2—O2—C18	117.2 (2)
H17A—C17—H17C	109.5	C2—O2—Dy1	114.15 (13)
H17B—C17—H17C	109.5	C18—O2—Dy1	127.57 (18)
O2—C18—H18A	109.5	C16—O3—Dy1	126.91 (15)
O2—C18—H18B	109.5	C15—O4—C17	117.4 (2)
H18A—C18—H18B	109.5	C15—O4—Dy1	116.98 (14)
O2—C18—H18C	109.5	C17—O4—Dy1	125.54 (18)
H18A—C18—H18C	109.5	N3—O5—Dy1	97.43 (18)
H18B—C18—H18C	109.5	N3—O7—Dy1	96.15 (16)
O1—Dy1—O3	74.18 (6)	N4—O8—Dy1	96.75 (16)
O1—Dy1—O10	95.32 (7)	N4—O10—Dy1	96.83 (16)
O3—Dy1—O10	146.76 (8)	N5—O11—Dy1	96.59 (15)
O1—Dy1—O8	142.24 (7)	N5—O13—Dy1	96.40 (14)

### Hydrogen-bond geometry (Å, °)

**Table:**

*D*—H···*A*	*D*—H	H···*A*	*D*···*A*	*D*—H···*A*
N2—H2···O3	0.85 (2)	1.87 (3)	2.570 (3)	139 (1)
C8—H8B···O12^i^	0.97	2.51	3.245 (3)	133
C10—H10···O5^ii^	0.93	2.32	3.076 (3)	138
C3—H3···O12^iii^	0.93	2.51	3.351 (3)	151
C7—H7···O9^iv^	0.93	2.56	3.376 (3)	147

Symmetry codes: (i) −*x*+1, −*y*+1, −*z*+1; (ii) −*x*+1, −*y*, −*z*+1; (iii) *x*−1/2, −*y*+1/2, *z*−1/2; (iv) *x*−1/2, −*y*+1/2, *z*+1/2.
